# Mechanical Characteristics of Hybrid Composites with ±45° Glass and 0°/90° Stainless Steel Fibers

**DOI:** 10.3390/ma11081355

**Published:** 2018-08-04

**Authors:** Caitlin O′Brien, Arash E. Zaghi

**Affiliations:** Civil and Environmental Engineering Department, University of Connecticut, 261 Glenbrook Road, Unit 3037, Storrs, CT 06269-3037, USA; arash.esmaili_zaghi@uconn.edu

**Keywords:** damage mechanics, hybrid Composites, mechanical properties, nonlinear behavior, energy dissipation, residual strain

## Abstract

Lack of energy dissipation is one of the shortcomings of conventional glass fiber reinforced composites. The addition of steel fibers to the conventional FRP composite to create a hybrid composite has been recently investigated as an option to address this limitation. The current literature is limited to composites reinforced with metal and non-metal fibers of the same alignment. In this study, hybrid and nonhybrid FRP composites of different layups, fiber content, and weave type were manufactured and subjected to hysteretic tensile loads. Woven glass fabrics in ±45° orientation were hybridized with unidirectional stainless steel fabrics in 0° and 90° orientations. This put the glass and steel layers in in-plane shear and normal stresses, respectively. The nonlinear stress–strain relationship, residual plastic strains, energy dissipation capability, and failure mechanisms of hybrid and nonhybrid composite type were compared. The hybrid composites presented improved energy dissipation, tensile strength, and stiffness when compared to nonhybrid ones. The applicability of an existing constitutive model that was originally developed for in-plane shear of conventional composites was investigated and refinements were proposed to present the hysteretic stress–strain relationship after addition of steel fibers. The refined model captured the increased plastic strain values and energy dissipation because of stainless steel fibers in the hybrid composite samples. An Armstrong–Frederick plasticity model was implemented to model the stress–strain relationship of the stainless steel composite samples.

## 1. Introduction

Fiber-reinforced polymer (FRP) composites are commonly comprised of glass or carbon fibers set in a resin matrix to form high-strength and stiffness material [1] whose behavior can be modeled using continuum damage mechanics (CDM). The brittle behavior of the conventional reinforcing fibers limits energy dissipation characteristics of composites [2]. Hybridization of these fibers with a ductile reinforcement has shown potential to address this shortcoming. However, the applicability of classic CDM models to predict the hysteretic stress–strain behavior and energy dissipation of hybrid FRP composites with ductile reinforcement has yet to be proven. Therefore, the objective of this study was to obtain experimental hysteresis stress–strain curves of hybrid fiberglass composites with ductile stainless steel reinforcement and accurately simulate these results using a CDM model that captures the energy dissipation of the entire hysteresis curve.

Common non-metal ductile fibers used in hybrid composites include polymeric fibers, like polypropylene (PP) [3,4], or natural fibers, like oil palm [5], silk [6,7,8], and bamboo [9,10]. Taketa et al. studied carbon fiber reinforced polypropylene (CFRPP) composites and CFRPP hybridized with self-reinforced polypropylene (SRPP). The CFRPP/SRPP hybrids had enhanced tensile failure strain with decreased tensile modulus and strength [4]. The hybridization of glass fibers and palm oil fibers resulted in enhanced elongation at break and the highest impact strength compared to the other nonhybrid composites in its study [5]. The mechanical properties of silk (biofiber) hybridized with glass [8] have also been studied and it was shown that the properties of glass/biofiber FRP composites were improved compared to the nonhybrid composites [8]. Studies by Thwe and Liao involved subjecting bamboo fiber reinforced polypropylene (BFRP) and bamboo-glass fiber reinforced polypropylene hybrid (BGRP) composites to hysteretic tensile loading after hygrothermal aging. The BGRP hybrids outperformed the BFRP composites in both fatigue resistance and retention of tensile stiffness and strength under environmental aging [9,10].

Although the hybridization with non-metal fibers increases elongation at break, fatigue performance, toughness, the stiffness of the composite is often compromised. In contrast, addition of metal fibers offers similar advantages as non-metal ductile fibers without compromising the stiffness of hybrid composites. The mechanical properties of quasi-unidirectional stainless steel composites have been investigated by various parties [11,12,13,14,15,16,17,18,19,20], and their mechanical properties show promise for the fibers to be incorporated into hybrid composites. These stainless steel fibers with diameters less than 30 microns have a strain-to-failure that is three to four times higher than a typical carbon or glass fiber composite with a modulus of elasticity as high as a high-modulus carbon fiber [15].

Performance characteristics of hybrid composites with steel and glass or carbon fibers have been investigated in several studies [18,21,22,23,24]. Mosleh et al. [22] showed that introducing steel fibers improves the impact resistance of carbon reinforced composites. McBride et al. [18] studied energy dissipation and residual strain characteristics of hybrid and nonhybrid unidirectional (UD) glass/steel composites under cyclic tensile loads. While certain hybrid layups had higher strength and energy dissipation compared to nonhybrid glass composites, failure strains were dominated by the UD glass fibers rather than reaching steel composite failure strains of 12%. Thysen [21] compared the properties of glass/steel composites with nylon and epoxy matrices and concluded that while the nylon composites have increased ductility, the epoxy composites have increased failure strains. Ahmed [24] concluded that the energy absorption of glass FRP composites under impact was increased with the addition of steel fibers. Hannemann et al. [23] cited improved electrical conductivity, impact and penetration resistance, and pseudo-ductile behavior in their study on UD and multiaxial hybrid carbon/steel FRP laminates. However, the scope of most of these studies has been limited to hybrid composites with UD fibers. It is hypothesized that placing the brittle and ductile fibers in the direction of loading prevents hybrid composites from reaching high strain-to-failure values, as the composite fails shortly after reaching failure strain of non-metal brittle fibers. Therefore, there is a need to test hybrid composites that allow the stainless steel fibers to reach their full failure strain potential, thus, maximizing energy dissipation.

The previous experimental results discussed guided this study to lay-up the glass fibers in ±45° and steel fibers in 0°/90° directions. Layers with ±45° fibers deform mainly in in-plane shear, where stress–strain behavior is nonlinear and energy dissipation and rupture strain values are significantly larger than composites with fibers in direction of loading. It is hypothesized that the combining the nonlinear behavior of layers reinforced with ±45° glass fibers with layers reinforced with steel fibers will utilize the large strain-to-failure property of steel fibers and increase energy dissipation characteristics.

Laminates with ±45° plies are modeled in CDM as an accumulation of permanent strain during in-plane loading and a degradation of the in-plane shear modulus, *G_12_*, a parameter obtained directly from experimental data following *ASTM D3518/D3518M-13, Standard Test Method for In-Plane Shear Response of Polymer Matrix Composite Materials by Tensile Test of a ±45° Laminate* [25]. Van Paepegem et al. [26,27] implemented this behavior in a constitutive model that simulates the backbone curve, or the outer envelope of the stress–strain curve, for glass FRP composites subjected to in-plane shear deformation. This mesoscale approach has proven advantageous, as multiple fiber weave types can be implemented in the same model.

Understanding stress–strain behavior plays a critical role in a material′s design and implementation, so the applicability of Van Paepegem′s model to capture the hysteretic stress–strain behavior of hybrid FRP composites with ductile steel fiber reinforcement in 0°/90° layup will be investigated. The mechanical performance of manufactured hybrid and nonhybrid glass FRP composite coupons with different glass and weave types will be evaluated with hysteresis experiments. The experimental data is transformed to obtain damage parameters that will be implemented in an altered version of Van Paepegem′s constitutive model that captures the energy dissipated during the unloading and reloading portions of hysteresis tests. How the additional plasticity of the steel in the hybrid composites affects the robustness of the model will be evaluated. The stress–strain relationship of unidirectional stainless steel FRP composites will be modeled using the Armstrong–Frederick kinematic hardening model [28,29], a well-known plasticity model.

## 2. Materials and Methods

A study was conducted to investigate the ability to model the mechanical performance of both hybrid and nonhybrid composites containing Type 316 stainless steel UD fibers in a 0°/90° layup and either 8H satin weave S-glass fibers or 4H modified twill weave E-glass fibers in a ±45° layup. The fiber volume fractions of the composites were determined theoretically and using thermogravimetric analysis (TGA). Mechanical properties of the composites were determined using hysteresis experiments. The mechanical performances were evaluated and damage parameters were obtained from each composite type.

### 2.1. Materials

#### 2.1.1. Reinforcing Fibers

Three types of reinforcing fibers were used to manufacture the composites: Type 316 stainless steel UD fibers, S-glass fibers with an 8H satin weave, and E-glass fibers with a 4H modified twill weave. The steel fibers were chosen for added energy dissipation while the glass fibers were chosen for their light weight, stiffness, and strength. The glass and weave type of the reinforcing fibers was varied to evaluate the mechanical performance of the hybrid composites with different woven geometries and the ability to implement these different layups in the CDM model.

##### Steel Reinforcement

**Figure 1:**
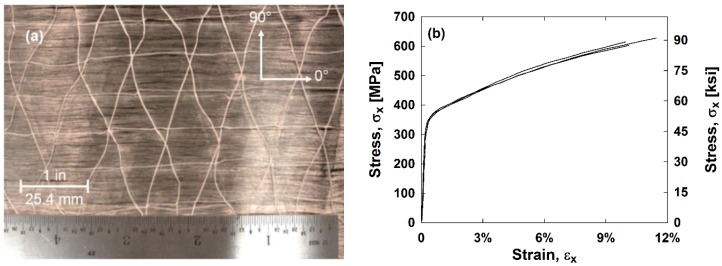
(**a**) Steel reinforcement fibers and (**b**) single stainless steel fiber stress–strain relationships.

The steel reinforcement used to manufacture the composite samples is a UD Type 316 stainless steel fiber weave provided by NV Bekaert SA (Kortrijk, Belgium) [30]. The weave has an areal density of 570 g/m^2^ (0.116 lb/ft^2^), and the fibers are a Type 316 stainless steel alloy with a diameter of 30 μm (0.001181 in). This particular fiber fabric was first manufactured for research purposes and has limited industry application. The weave is shown in Figure 1a. Polyethylene succinate (PES) cross yarns with an average diameter of 15 μm (0.000590 in) maintain the integrity of the weave in the warp (0°) direction without contributing significantly to the mechanical properties of the fabric. The fiber manufacturing process uses a bundle drawing technique.

The mechanical properties of the fibers were obtained using single fiber testing in a dynamic mechanical analyzer (DMA) system, a Texas Instruments (Dallas, Texas, USA) DMD Q800. The samples had an average Young′s modulus of 193 GPa (28,000 ksi), a yield stress of 365 MPa (53 ksi), an ultimate tensile strength of 620 MPa (90 ksi), and a failure strain of 11%. The longitudinal stress–strain relationships are in Figure 1b.

##### Fiberglass Reinforcement

The fiberglass fibers used to manufacture the composite samples were both woven E-glass and S-glass fibers obtained from Fiber Glast Developments Corporation (Brookville, OH, USA). The E-glass is a 4H modified twill weave with warp and fill yarns in the 0 and 90° directions. The fabric has an areal density of 288.2 g/m^2^ (0.0590 lb/ft^2^) and a material density of 2.54 g/cm^3^ (0.0001585 lb/ft^3^). E-glass typically has a Young′s modulus between 72 and 85 GPa (10,400 and 12,300 ksi). Twill weaves offer greater conformability and slightly higher strength when compared to plain weave fabrics.

The S-glass is an 8H satin weave, which is flatter than other weave types and conforms to shapes easily. The fabric has an areal density of 300.1 g/m^2^ (0.06146 lb/ft^2^) and a material density of 2.530 g/cm^3^ (0.0001580 lb/ft^3^). S-glass typically has a Young’s modulus between 86 and 93 GPa (12,500 and 13,500 ksi). Each side of the fabric is either mostly fill or warp fibers, so the half of the plies must be inverted to make a symmetrical laminate.

#### 2.1.2. Resin Matrix

The matrix used in the manufacturing of the composites is a two-part thermosetting system comprised of a resin and hardener. EPON 828, the resin, is a difunctional bisphenol A/epichlorohydrin derived liquid epoxy resin. EPIKURE 3055, the curing agent, is an aliphatic amine hardener. Both were supplied by Hexion (Columbus, OH, USA). The manufacturer-recommended resin-to-hardener weight ratio of 2:1 was used to obtain optimal polymer cross-linking. EPON 828 is a commonly used industry resin due to its mechanical versatility and high chemical resistance [31]. EPIKURE 3055 hardener has a low viscosity and extended pot life, ensuring workability of the matrix and faster impregnation of the fibers during manufacturing [32]. Tensile hysteresis experiments following American Society for Testing and Materials (ASTM) D638 [33] was performed by McBride et al. [18] on epoxy dog bones to characterize the mechanical properties. The elastic modulus is 2.88 GPa (417.7 ksi), the ultimate tensile strength is 55.53 MPa (8.054 ksi), and the failure strain is 3%.

### 2.2. Manufacturing of Composite Specimens

Five composite plates were manufactured with different layups and fiberglass and steel contents. Their layups are outlined in Table 1, with composites classified as hybrids containing fiberglass and stainless steel fibers. In the layup column, a “G” represents the sample′s respective glass type and an “S” represents steel. The superscript following the composites ply material type is the layup angle of the ply in degrees. The 0° ply direction corresponds to the longitudinal, x-axis; also the direction of loading during tensile testing. A numerical subscript outside of brackets represents the number of layers in this layup and an “s” subscript outside of brackets indicates that the layup is symmetric about the laminate midplane. The hybrid composites were manufactured with a targeted fiberglass-to-steel ratio of 70 to 30, which McBride et al. [34] found to have the best performance in comparison to other fiberglass-to-steel ratios.

The stainless steel in the hybrids had 0 and 90° plies to investigate obtaining further energy dissipation from the steel.

The composites were manufactured using a vacuum bagging process. For each ply layer, a 304 × 304 mm (12 in × 12 in) square was cut from the fiber′s respective roll at the fiber angle desired. The epoxy was mixed with a 2:1 resin-to-hardener weight ratio, and it was ensured minimal air bubbles were mixed into the system. These plies were laid up by hand. Then, the layup was placed into a vacuum bag configuration where the laminate was subjected to a vacuum pressure until the resin is cured. Vacuum bagging prevents excess resin, air, and humidity from entering the laminate. This improves fiber-to-resin ratio, a key component in maximizing the strength-to-weight ratio of the composite.

Once demolded, the plate was cut into 25.4-mm (1-in) wide coupons. To avoid premature failure due to stress concentrations in the testing grips, 50.8-mm (2-in) long tabs were applied to the ends of the coupons. The end tabs were G10 fiberglass, an industrial laminate made from glass fabric embedded in epoxy resin. The G10 was beveled and applied to the ends of the samples with a Loctite (Düsseldorf, Germany) Armstrong EA E-120HP epoxy adhesive resin.

The volume fraction (*v_f_*) was calculated using Equation (1) and is based on the composite thickness (*t*), material density (*ρ*), number of fiber layers (*n*), and fabric area density (*A*). The average thickness, length, and the theoretical fiber volume fractions of the composites are in Table 2.

(1) $$v_{f}=\left(n*A\right)/\left(\rho *t\right)$$

The volume fraction of the composites was also found using TGA. Small samples of each composite were subjected to high temperatures to decompose the composite’s organic resin. The weight remaining is considered the remaining fibers. Using the remaining weight and the known densities of the materials, the fiber volume fraction can be found. These values are also in Table 2 and are comparable to theoretical values.

### 2.3. Experimental Methodology

Hysteresis experiments were performed on each specimen to obtain the shape of the hysteresis loops and energy dissipation. All testing was performed using an MTS (Eden Prairie, MN, USA) Exceed Model E45 electromechanical universal testing frame with a maximum load capacity of 100 kN (22480 lb). The hysteresis experiments involved applying a tensile machine displacement at 0.1524 mm/s (0.006 in/s) until a specified strain was reached. Then, the sample was unloaded to 0.22 kN (50 lb) at the same rate. Each subsequent loading cycle increased by a specified strain and then unloaded to 0.22 kN (50 lb) to maintain grip pressure. The strains for each cycle were the following: 0.25%, 0.5%, 0.75%, 1%, 2%, 3%, 4%, 6%, 8%, and 10%. Multiple cycles were implemented at strains less than 1% in order to effectively capture the stress–strain response changing from elastic to plastic. The longitudinal strain was measured using an Epsilon Technology Corporation (Jackson, WY, USA) model 3542 axial clip-on extensometer with a 50.8-mm (2-in) gage length. Digital image correlation (DIC) was utilized to obtain strain distributions. A DIC system requires a speckle pattern to be applied to the face of each sample using a textured spray paint. The DIC image data was recorded at a rate of 2 frames per second (fps) using two Point Grey (Richmond, BC, Canada) Grasshopper3 50S5M-C USB3 cameras.

## 3. Results and Discussion

### 3.1. Tensile Properties

**Figure 2:**
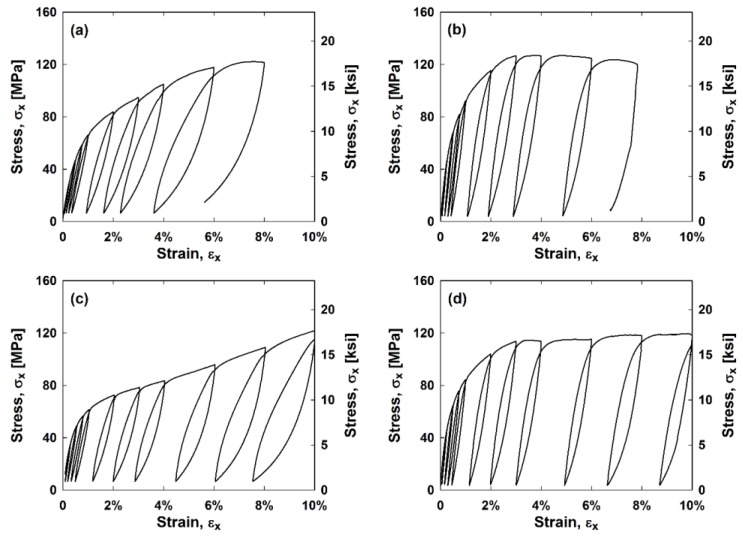
Average (**a**) E-glass; (**b**) E-glass hybrid; (**c**) S-glass; and (**d**) S-glass hybrid composite stress–strain curves.

**Figure 3:**
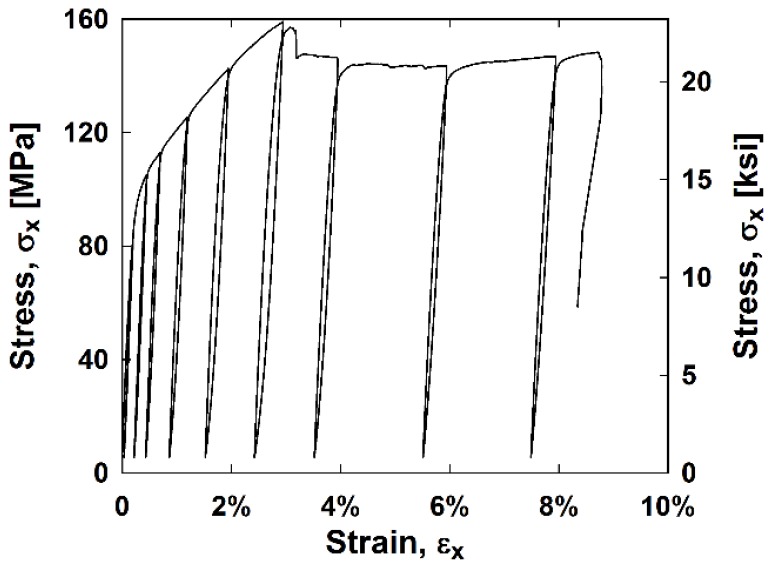
Average stainless steel composite stress–strain curves.

The average longitudinal stress, *σ_x_*, versus longitudinal strain, *ε_x_*, hysteresis curves for the hybrid and nonhybrid composites are in Figure 2. The average hysteresis curves for the stainless steel composites are in Figure 3. It can be observed that stainless steel has the highest stiffness and strength of all the samples while the all glass composites exhibit the lowest stiffness and strength. All samples except for the S-glass sample failed before reaching 10 cycles. Each composite’s stress–strain curve has a “knee shape” at approximately 3% strain, the failure strain of the matrix.

**Figure 4:**
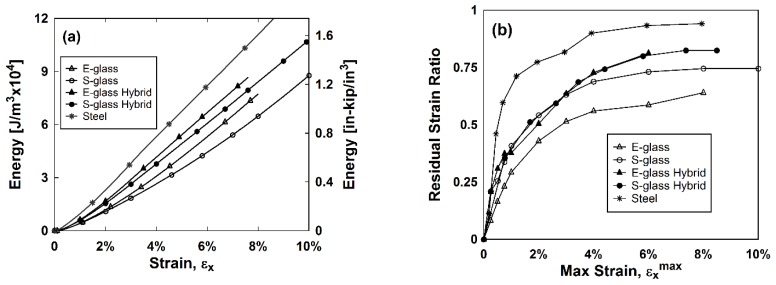
(**a**) The energy dissipated versus the maximum strain for each cycle and (**b**) the residual strain ratio versus the maximum strain for each cycle.

Further exploration into the mechanical performance of these composites can be seen in the energy curve up to 10% strain in Figure 4a. The x-axis represents the maximum strain reached at each cycle prior to unloading. The y-axis represents the energy dissipated during each loading cycle found by integrating the stress–strain relationship. These relationships were averaged due to the similarity of results between samples of the same type. The stainless steel composite dissipated the most energy followed by the two hybrids, which both dissipated a similar amount of energy. The E-glass and S-glass composites dissipated the least amount of energy, however, the S-glass composite reached the highest strains.

The average residual strain ratio versus the maximum strain was plotted (Figure 4b). The residual strain ratio is the ratio of the strain reached at the end of unloading and the strain reached prior to unloading. The lower the residual strain ratio, the more stable the material is and the better capability the material has to recenter. Recentering indicates the ability of a material to stabilize itself and recover from displacements following traumatic events like earthquakes. As expected, the stainless steel composite had the highest residual strain ratio due to its inelastic behavior. The E-glass composite had the lowest residual strain ratio while the S-glass composite performed similarly to the two hybrids until around 3% strain, the failure strain of the matrix.

### 3.2. Failure Mechanisms

**Figure 5:**
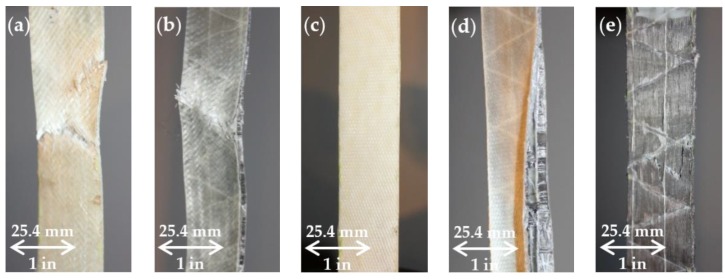
(**a**) E-glass; (**b**) E-glass hybrid; (**c**) S-glass; (**d**) S-glass hybrid and (**e**) Stainless steel composite failure modes.

Different failure mechanisms were exhibited for each specimen type, depicted in Figure 5. Matrix cracking was visually observed in the hybrid and nonhybrid samples. The E-glass composites failed by fiber rupture. The E-glass hybrid failed via fiber rupture as well, but also exhibited some delamination between plies along the free edge. The S-glass composites never completely failed. Instead, they were stretched into a “rope-like” material. The S-glass hybrid’s plies delaminated along the free edge, which was the samples′ resulting failure mode. The delamination occurred between the stainless steel plies, which may be due to a bonding issue with the stainless steel fibers and the epoxy, as well as interlaminar stresses at the free edge. The stainless steel composites had a fiber rupture failure mode. The longitudinal strain at which the stainless steel samples failed varied from 3% to 6%, indicating a failure to obtain the full strain potential of the stainless steel fibers.

### 3.3. Modelling Hysteresis Experiments

Two different models are implemented to simulate the stress–strain behavior of the composites. First, a continuum damage mechanics (CDM) model originally implemented by Van Paepegem et al. [26,27] is used to model the stress–strain relationship of the hybrid and nonhybrid glass FRP composites. Then, the UD stainless steel composites′ stress–strain curve is modeled using the Armstrong–Frederick kinematic hardening model [28,29].

#### 3.3.1. Hybrid and Nonhybrid Glass Composite Model

It has been well studied that when placed in in-plane shear, FRP composites exhibit a nonlinear stress–strain behavior. Furthermore, this nonlinear stress–strain relationship can be modeled using constitutive equations based on CDM. First, shear stress and shear strain of the hybrid and nonhybrid glass FRP composites will be found using stress and strain transformations and following ASTM D3518/D3518M-13, Standard Test Method for In-Plane Shear Response of Polymer Matrix Composite Materials by Tensile Test of a ±45° Laminate [25]. Then, curve-fitting relationships originally developed by Van Paepegem et al. [26,27] will be generated from the transformed experimental data to create constitutive relationships that models the nonlinear stress–strain behavior. This model is expanded to also capture the unloading/reloading portions of the hysteretic tensile results. The ability for the expanded model to capture this additional energy dissipation is evaluated by simulating the experimental stress–strain results in a MATLAB^®^ code.

According to 2-dimensinal lamina analysis of laminates subjected to plane stress, laminates with ±45° plies have shear stress that depends only on the axial tensile stress. Therefore, the shear stress in the material coordinates, *τ_12_^i^*, is independent of the material properties and is calculated as:

(2) $$\tau_{12}^{i}=\frac{F^{i}}{2A_{c}}$$

where *F* is the load applied to the test coupon at point *i* during loading and *A_c_* is the cross sectional area of the composite sample. According to strain transformation relations, the shear strain, *γ_12_*, in material coordinates can be found by transformation of the axial and transverse strains, *ε_xx_* and *ε_yy_*. The axial and transverse strains used to calculate the shear strain were obtained from the DIC strain distribution data. For orthotropic laminates under tension, the shear strain in the specimen axis, *γ_xy_*, is zero and independent of *γ_12_*. The equation for shear strain in the material directions, *γ_12_^i^*, at each point *i* is as follows:

(3) $$\gamma_{12}^{i}=\varepsilon_{xx}^{i}-\varepsilon_{yy}^{i}$$

Once the shear stress–strain relationship is found, relationships that describe the nonlinear behavior can be developed. The initial inplane shear properties degrade when being subjected to increasing strains. This behavior can be classified using two variables: the shear damage, *D_12_*, and the permanent shear strain, *γ_12_^p^*. The shear stress, *τ_12_*, is defined using the following constitutive relationship with shear strain, *γ_12_*:

(4) $$\tau_{12}=G_{12}^{0}\times \;\left(1-D_{12}\right)\left(\gamma_{12}-\gamma_{12}^{p}\right)$$

where the difference between the total strain and the unrecoverable permanent strain, *γ_12_^p^* is the elastic strain, *γ_12_^e^*:

(5) $$\gamma_{12}^{e}=\gamma_{12}-\gamma_{12}^{p}$$

**Figure 6:**
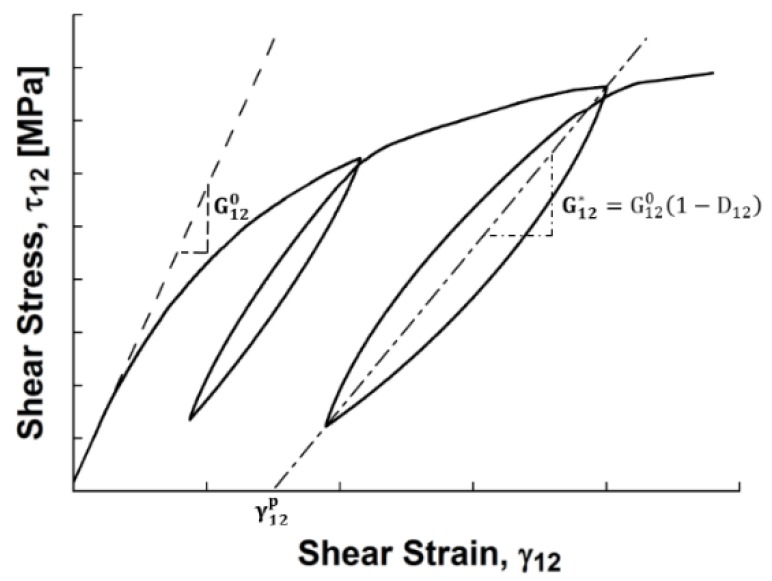
Definition of initial and damaged shear modulus and permanent strain.

The initial shear modulus, *G_12_^0^*, decreases when being subjected to increasing strains. This “damaged” shear modulus, G_12_^*^, is calculated as the slope between the stress and strain at a fully loaded cycle and the stress and strain at the same cycle fully unloaded. The damaged shear modulus is shown in Figure 6. The relationship between the initial and damaged shear modulus is represented by the shear damage, *D_12_*:

(6) $$D_{12}=1-\frac{G_{12}^{*}}{G_{12}^{0}}$$

How much damage the shear modulus accumulates varies from 0 to 1, zero being not damaged, and 1 being completely damaged. The permanent shear strain is the unrecoverable shear strain at the end of each unloading cycle and can be found by finding the x-intercept of the damaged shear modulus line. These relationships were originally developed by Lafarie–Frenot [35] when they studied the in-plane shear behavior of long carbon-fiber composites. A representation of obtaining the initial shear modulus, the damaged shear modulus, and the permanent shear strain is depicted in Figure 6.

Once the damage parameters are found for each cycle, they can be plotted and curve fitted using Equations (7) and (8) so they can implemented in the constitutive shear stress equation (Equation (4)). Two curve fitting equations were developed by plotting the permanent shear strain versus maximum strain and plotting the shear damage versus elastic strain. The MATLAB^®^ (Natick, MA, USA) Curve Fitting Toolbox was utilized to obtain the equations for these curves. The magnitude of the permanent shear strain as a function of total strain was fitted with the following equation:

(7) $$\left|\gamma_{12}^{p}\left(\gamma_{12}\right)\right|=\frac{-\ln\left(1-a\times \;b\times {\left|\gamma_{12}\right|}^{1.5}\right)}{b}$$

where *a* and *b* are curve fitting constants obtained for each composite type. The shear damage curve fitting equation as a function of elastic strain is as follows:

(8) $$D_{12}\left(\gamma_{12}^{e}\right)=c\left(\exp\left(d\times \;\left|\gamma_{12}^{e}\right|\right)-\exp\left(e\times \left|\gamma_{12}^{e}\right|\right)\right)$$

where *c*, *d*, and *e* are curve fitting constants obtained for each composite type. The constants obtained from Equations (7) and (8) are summarized as the average of two of each type of composite in Table 3.

**Figure 7:**
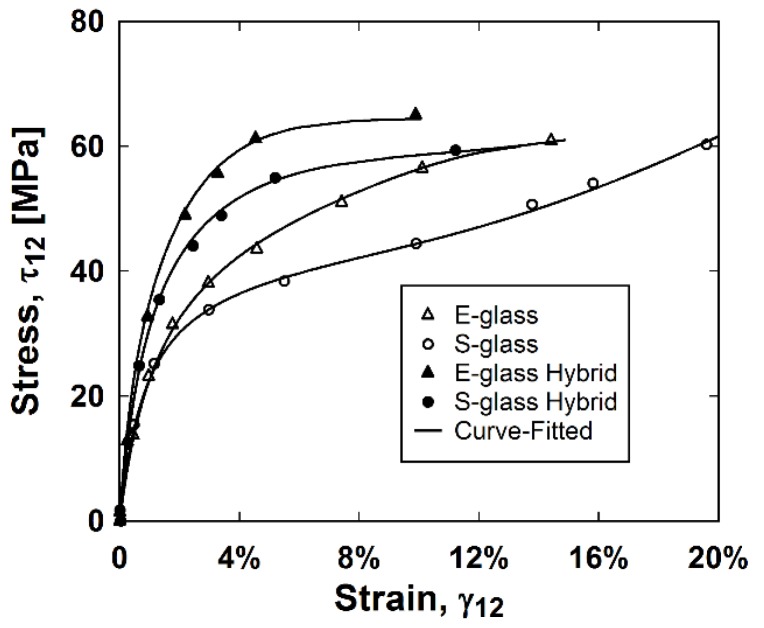
Experimental and curve-fitted shear stress–shear strain relationships.

Utilizing the curve fitting Equations (7) and (8), as well as the shear stress Equation (4), the stress–strain backbone relationships can be found for each sample. The averaged experimental and simulated shear stress versus shear strain backbone relationships are in Figure 7.

It is suggested by Kellas et al. [36] that a fiber rotation of 1° occurs for every 2% axial strain or 3.5% shear strain. If this fiber scissoring continues while being unbounded, the assumptions made for a ±45° laminate are no longer valid. Therefore, the ASTM D3518/D3518M-13 [25] test method specifies the termination of data reporting after 5% calculated shear strain, limiting fiber scissoring by 1.5°. To investigate modeling the entire shear stress–strain behavior, the backbone relationships were simulated for all stresses and strains.

The accuracy of the initial portion of the backbone relationship was increased by including numerous cycles in the experiments before 1% strain because the equations to simulate the damage parameters were sensitive to early strains. The curve fitting parameters of composites of the same type were similar, indicating reliability and repeatability of the curve-fitting process. As reflected in the curve fitting parameters in Table 3 as well as the backbone relationships in Figure 7, the constants make meaningful insights into the composite behavior at the range of strains these equations were fitted for. The added stiffness and elasto–plastic behavior of the stainless steel fibers is clear in the difference in curve-fitting values between the hybrid composites and their respective nonhybrids. The hybrids accumulated more permanent strain than the nonhybrid composites, as reflected by the less negative *b* constants. Constants *e* and *d* adjust the amount of damage accumulated in lower and higher strains, respectively. As indicated by the *e* constants, the stainless steel fibers caused the hybrid composites to accumulate more damage at lower elastic strains. The *e* constants adjust how the shear damage curve transitions from negative to positive curvature, thus, how quickly the damage parameter reaches 1 at higher strains. This parameter, however, depends more on the range of elastic strains the shear damage was fitted for.

#### 3.3.2. Unidirectional Stainless Steel Composite Model

Due to their inherent behavior, the UD stainless steel composites could not be accurately modelled using the CDM constitutive equations that describe the hybrid and nonhybrid glass FRP composites. Therefore, the Armstrong–Frederick kinematic hardening model was implemented to simulate the stress–strain behavior of the UD stainless steel composites. This well-known model introduces nonlinearity to the kinematic hardening rule. First, the basics of nonlinear kinematic hardening are described. Then the pseudocode of the model′s implementation in MATLAB^®^ is discussed. Experimental backbone stress–strain results of the UD stainless steel composites are then simulated using the MATLAB^®^ code.

The Armstrong–Frederick model is based on the basic principles of the classical theory of plasticity: the decomposition of the total strain into an elastic and plastic part, the yield criterion, the flow rule, and the hardening rule. The total strain is elastic and fully recoverable in the elastic region of loading. Once the elastic limit is exceeded, the loading is elastoplastic and the total strain is the sum of the elastic strain, *ε^e^*, and the unrecoverable plastic strain, *ε^p^*:

(9) $$\varepsilon =\varepsilon^{e}+\varepsilon^{p}$$

The yield criterion determines whether the material is elastic or inelastic at each stress increment. The Von–Mises condition, *f,* is commonly used yield criterion. For an isotropic homogeneous material, Von-Mises is described as:

(10) $$f=\sqrt{\frac{3}{2}\left(s-a\right):\left(s-a\right)}-\sigma_{Y}$$

where *σ_Y_* is the yield stress of the material, ***s*** is the deviator of the stress tensor, ***σ***, and ***a*** is the deviator of the backstress, ***α***. In the principal coordinate system, the Von–Mises condition creates an elliptical yield surface. The hardening rule describes the change of shape and location of the yield surface. In the case of kinematic hardening, the size of the initial yield surface remains the same, but the center of the ellipse is shifted. The backstress, *α,* is the coordinates of the center of the ellipse. The change in backstress, *dα*, is calculated as:

(11) $$d\alpha =\frac{2}{3}Cd\varepsilon^{p}-\gamma \alpha dp$$

where *C* and *γ* are material parameters and *dp* is the increment of accumulated plastic strain:

(12) $$dp=\sqrt{\frac{2}{3}Cd\varepsilon^{p}:d\varepsilon^{p}}$$

The plastic deformation development, *dε^p^*, is directed by the plastic flow rule, which governs the plastic strain increment through the use of a plastic multiplier, *dλ*:

(13) $$d\varepsilon^{p}=\sqrt{\frac{3}{2}}d\lambda \frac{\partial f}{\partial \sigma }$$

In the case of uniaxial loading, Equation (10) can be simplified:

(14) $$f=\left|\sigma -\alpha \right|-\sigma_{Y}=0$$

Taking plastic incompressibility into account, Equation (11) is simplified to consider only its deviatoric portion:

(15) $$d\alpha =Cd\varepsilon^{p}-\gamma \alpha \left|d\varepsilon^{p}\right|$$

Now that the governing equations of the Armstrong–Frederick model have been established, the pseudocode of the MATLAB^®^ code used to simulate experimental stress–strain curves of the UD stainless steel composites can be described. First, the elastic stress is predicted using the total stress from the previous increment, *σ*, Young′s modulus, *E*, and total strain increment, *dε*:

(16) $$\sigma^{e}=\sigma +Ed\varepsilon$$

Equation (14) is calculated to determine whether the composite is in the elastic phase or plastic phase. If *f* ≈ 0, the phase is elastic and the total stress for the current increment is equal to the elastic stress calculated using Equation (17). Otherwise, the phase is plastic and the elastic back stress, *σ^eb^,* is predicted:

(17) $$\sigma^{eb}=\sigma^{e}-\alpha$$

This elastic back stress is used to calculate the plastic multiplier increment, *dλ*:

(18) $$d\lambda =\frac{\sigma^{eb}-\psi \sigma_{Y}}{\psi \left(E+C\right)-\gamma \alpha }$$

where *ψ* = ± 1 based on the sign of *dε*. The plastic strain increment is found as the product of the plastic multiplier increment and the sign of *dε*, *ψ*, and is substituted into Equation (15) to find the change in backstress. The total backstress, *α*, is:

(19) α=α+dα

Lastly, the total stress when the loading is in plastic phase is calculated as:

(20) $$\sigma =\psi \sigma_{Y}+\alpha$$

**Figure 8:**
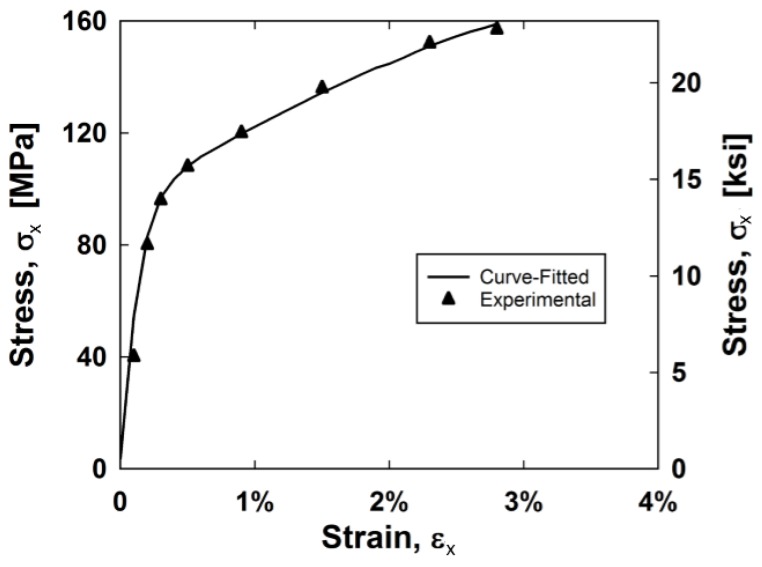
Experimental and curve-fitted shear stress–shear strain relationships.

This pseudocode was implemented for the stainless steel composite backbone curve up to 3% strain. The three material constants, *E, σ^Y^, C,* and *γ* are 51,850, 90, 5000 MPa, and 0.000055 MPa, respectively. The results are displayed in Figure 8.

### 3.4. Simulated Hysteresis Behavior

#### 3.4.1. Hybrid and Nonhybrid Glass Composite Model

**Figure 9:**
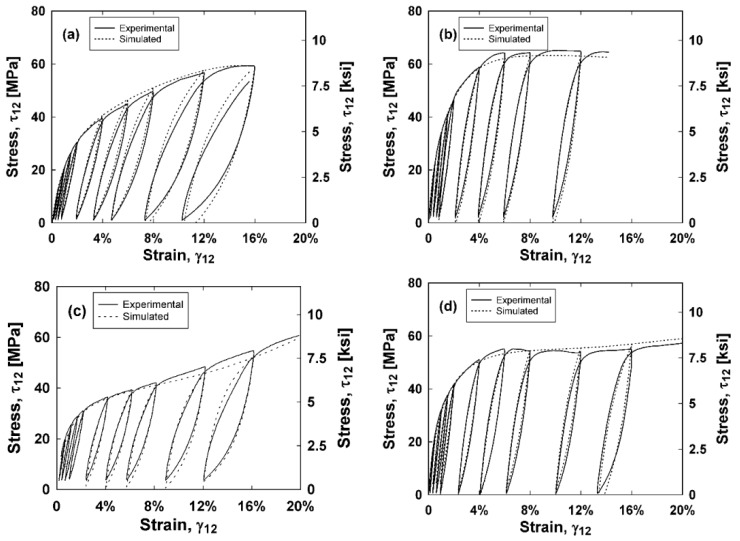
Experimental and simulated (**a**) E-glass; (**b**) E-glass hybrid; (**c**) S-glass; and (**d**) S-glass hybrid composite shear stress–shear strain curves.

**Figure 10:**
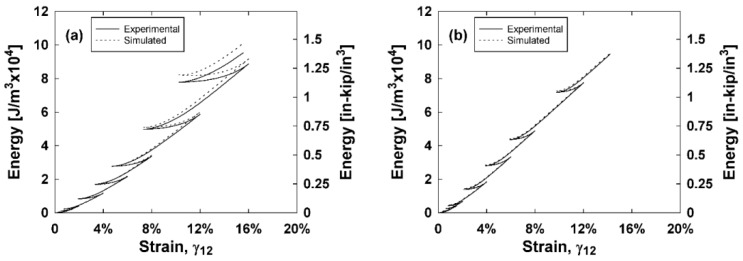

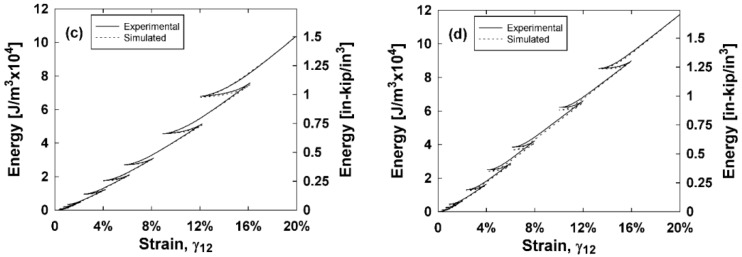
Experimental and simulated (**a**) E-glass; (**b**) E-glass hybrid; (**c**) S-glass; and (**d**) S-glass hybrid energy dissipated versus shear strain curves.

To investigate the ability for the constitutive model to predict the composite hysteresis behavior and energy dissipation, a MATLAB^®^ code was generated to simulate the shear stress versus shear strain curves. These simulated curves are integrated to obtain the energy dissipated of each composite. Inputs of the MATLAB^®^ code include the experimental shear strains and the damage parameters for each composite type. The sign of the change in strain for each step, as well as the maximum strain reached up to the current step of the simulation determined whether the current step was loading, unloading, or reloading. A positive change in strain and a strain that exceeds the current maximum strain indicates a shear stress that follows the stress–strain backbone curve. Damage parameters are calculated using Equations (7) and (8) and the shear stress is found using Equation (4). A strain that is less than the current maximum strain and a negative change in strain indicates that there is unloading. One can assume that the unloading curve is symmetric to the backbone curve. To simulate this, the total strain is zeroed at the start of unloading. Then, the subsequent changes in strains are halved and added to the initially zeroed strain. Damage parameters are calculated using Equations (7) and (8) and the calculated stresses found using Equation (4) are doubled and subsequently subtracted from the final stress on the backbone curve before unloading. By halving and doubling the strains and stresses, respectively, this unloading curve is symmetric. Lastly, a strain that is less than the current maximum strain and a positive change in strain indicates that there is reloading. The reloading portion is calculated similarly to the unloading portion, except the doubled stresses are subsequently added to the last stress obtained during unloading. It is reasonable to assume that the unloading and reloading portions are not perfectly symmetric. Instead of doubling the calculated stresses, a symmetry factor between 1.75 and 2 is used to multiply the stress by to find a good fit for the unloading portions of the curves. These values are 1.8, 1.8, 2, and 1.75 for the E-glass, E-glass hybrid, S-glass, and S-glass hybrid composites, respectively. This MATLAB^®^ code was implemented for each composite type and the results are compared with experimental stress–strain curves in Figure 9. Both the experimental and simulated curves are integrated to find the energy dissipated and these results are plotted against the shear strain in Figure 10.

As indicated by Figure 9 and Figure 10, the unloading and reloading portions of the experimental stress–strain curves are accurately simulated using the MATLAB^®^ code, thus also accurately predicting the energy dissipated for each composite type. The symmetry factors were chosen to best represent the experimental data as a whole, and tend to simulate less accurate results at higher strains.

#### 3.4.2. Unidirectional Stainless Steel Composite Model

**Figure 11:**
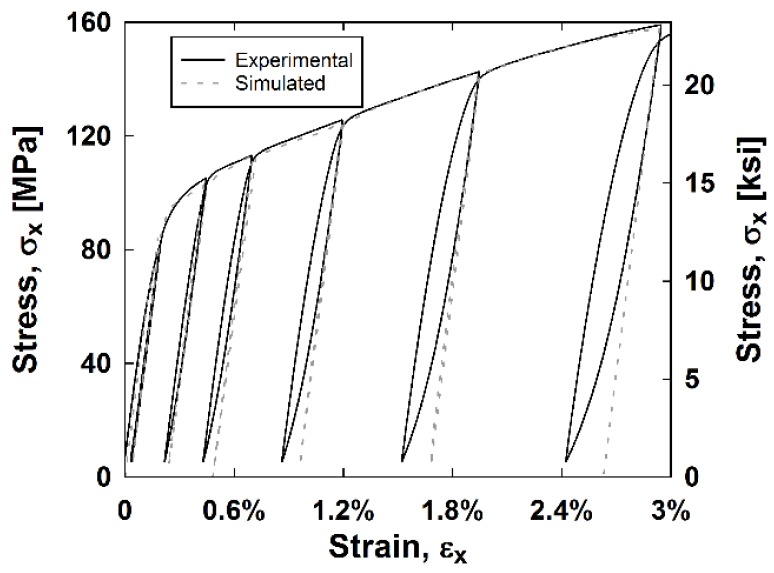
Stainless steel composite experimental and simulated stress–strain curve.

The stainless steel composite′s hysteresis curve is simulated in MATLAB^®^ similarly to the CDM model. The unloading and reloading portions of the curves have strains that are halved and stresses that are doubled, respectively, so the unloading portion is symmetric. However, since there was little curvature in the hysteresis loops of the stainless steel composite, the unloading and reloading change in strain of the curve are multiplied by the stiffness of the material, *E*, creating linear changes in stress while unloading and reloading. Results from this simulation in MATLAB^®^ are depicted in Figure 11. As indicated in Figure 11, the simulated results get less accurate at higher hysteresis cycles and unloading curves are over predicted. However, the backbone curve and simulated results at lower strains are accurate.

## 4. Conclusions and Future Work

This study investigated the mechanical performance of both hybrid and nonhybrid composites containing Type 316 stainless steel UD fibers in a 0°/90° layup and either 8H satin weave S-glass fibers or 4H modified twill weave E-glass fibers in a ±45° layup. The results of this study gave rise to the following findings:

- Hybrid composites with glass and steel fibers had higher energy dissipation, stiffness, and strength when compared to their respective nonhybrid E-glass and S-glass composites;
- The hybrid composites with woven fiberglass subjected to in-plane shear in this study outperformed the UD hybrid composites tested by McBride et al. [18] in terms of energy dissipation and strain at failure;
- Geometry of woven materials is an important consideration when designing composites. The 4H modified twill weave outperformed the 8H satin weave in energy dissipation and had a lower residual strain ratio, thus reaching higher elastic strains;
- The S-glass composites did not reach their full shear strength potential, as the matrix was most likely too weak to support the higher strength of the S-glass fibers;
- The steel fibers did not reach their ultimate strain potential and their delamination from the composite was a major contribution to the failure mode of the hybrid samples. Fiber–matrix interface is an important consideration when designing a composite with desired mechanical properties. Callens et al. [17] improved the mechanical performance of their stainless steel fiber composites by modifying the adhesion of the stainless steel fibers with different silane treatments. The application of modified adhesion and use of a tougher matrix will be considered in future manufacturing of hybrid composites;
- In future work, the damage and failure modes of the hybrid composites should be investigated further. Jalalvand et. al [37] investigated the different damage modes of hybrid composites comprised of high strain material plies and low strain material plies as a function of absolute and relative thickness of the low strain material. By knowing the damage mode of a particular layup, one can improve the design and function of the composite;
- The nonlinear behavior of the composites when subjected to in-plane shear can be described as a degradation of the shear modulus and accumulation of permanent strain. The inelastic behavior of the stainless steel fibers altered the damage behavior of the hybrid composites compared to the all glass composites. The constitutive model developed in this study was robust enough to accurately model the additional plasticity that the stainless steel fibers contribute to the hybrid composites. The MATLAB^®^ code described accurately predicts the hysteresis experiments as well as the energy dissipated in the experimental results. The Armstrong–Frederick model was able to accurately predict the behavior of the stainless steel fibers;
- The ability for one to investigate different hybrid composites designs by simulating their behavior is invaluable. Therefore, in future work, comparing superpositions of the steel fiber layers with the fiberglass layers will be investigated. By combining the Armstrong–Frederick model and the CDM model in finite element modelling (FEM), one can simulate different layups of the composites.

## Figures and Tables

**Table 1:** Composition of prepared composites.

Composite	Composition	Layup
E-glass	6 layers G^±45^	[G^±45^]_3s_
S-glass	6 layers G^±45^	[G^±45^]_3s_
Stainless Steel	6 layers S^0^	[S^0^]_3s_
E-glass Hybrid	6 layers G^±45^, 2 layers S^0^, 2 layers S^90^	[G^±45^ G^±45^ S^0^ S^90^G^±45^]_s_
S-glass Hybrid	6 layers G^±45^, 2 layers S^0^, 2 layers S^90^	[G^±45^ G^±45^ S^0^ S^90^G^±45^]_s_

**Table 2:** Average composite thicknesses and fiber volume fractions.

Sample	Thickness [mm (in)]	Length [cm (in)]	Glass Fiber Fraction [Theoretical % (TGA %)]	Steel Fiber Fraction [Theoretical % (TGA %)]	Total Fiber Volume Fraction [Theoretical % (TGA %)]
E-glass	1.3 (0.0505)	17.03 (6.706)	51.08 (55.81)	--	51.08 (55.81)
S-glass	1.45 (0.0571)	17.14 (6.75)	49.75 (52.82)	--	49.75 (52.82)
Stainless Steel	1.592 (0.0627)	19.05 (7.5)	--	27.38 (36.52)	27.38 (36.52)
E-glass Hybrid	2.277 (0.08963)	17.86 (7.031)	31.566 (32.98)	12.67 (13.31)	45.28 (46.29)
S-glass Hybrid	2.486 (0.0979)	16.51 (6.5)	26.90 (32.81)	10.80 (13.13)	40.69 (45.94)

**Table 3:** Average curve fitted parameters for each composite type.

Composite	G_12_^0^ [MPa]	Permanent Strain			Shear Damage	
		a	b	c	d	e
E-glass	3624	2.366	−8.574	0.6979	1.112	−50.74
S-glass	4891	2.868	−6.968	0.7170	1.432	−93.02
E-glass hybrid	7900	2.743	−3.360	0.4605	15.62	−199.6
S-glass hybrid	6603	3.052	−5.018	0.5326	9.995	−111.2

## Founding

The work at the University of Connecticut leading to this publication has received funding from PFI: AIR-TT National Science Foundation Grant #1500293.
